# Approaches Adopted by Researchers to Measure the Quality of the Experience of People Working from Home: a Scoping Review

**DOI:** 10.1007/s41347-022-00264-4

**Published:** 2022-07-06

**Authors:** Stefano Federici, Maria Laura De Filippis, Maria Laura Mele, Simone Borsci, Marco Bracalenti, Giovanni Bifolchi, Giancarlo Gaudino, Massimo Amendola, Antonello Cocco, Emilio Simonetti

**Affiliations:** 1 Department of Philosophy, Social and Human Sciences and Education, University of Perugia, Perugia, Italy; 2 Department Learning, Data Analysis, and Technology, Cognition, Data and Education CODE Group, Faculty of Behavioural, Management and Social Sciences, University of Twente, Enschede, the Netherlands; 3 Department of Surgery and Cancer, Faculty of Medicine, NIHR London IVD, Imperial College, London, UK; 4 DGTCSI-ISCTI, Directorate General for Management and Information and Communications Technology, Superior Institute of Communication and Information Technologies, Ministry of Economic Development, Rome, Italy; 5 Department of Public Service, Prime Minister’s Office, Rome, Italy

**Keywords:** Experience, Satisfaction, Workers’ experience, Working from home, Remote working, Assessment

## Abstract

Working from home (WFH) remotely is a modality of working that requires the careful design of systems of rules and tools to enable people to exchange information and perform actions. WFH is expected to expand after the COVID-19 pandemic. How to assess and compare in a reliable way the experience of workers with different (sociotechnical) systems of WFH is a central point to supporting the diffusion of acceptable modalities of working. However, the concept of experience and how it can be measured in the domain in WFH is yet to be clearly characterized. Following the Preferred Reporting Items for Systematic Reviews and Meta-Analyses (PRISMA) methodology for scoping reviews, we systematically map the approaches used by researchers to assess WFH, identify which aspects are usually investigated, and examine how such aspects are usually measured in terms of questions and tools. Literature is collected using Scopus and Web of Science. Thirty-four records out of 323 focusing either on validating a scale, presenting theoretically the experience of workers or testing this empirically are included in the qualitative synthesis. The results highlight a lack of unified terminology and tools, with assessments of workers’ experience mainly characterized by survey approaches and qualitative questions. Clustering together the most investigated aspects in the literature and reviewing how these aspects are assessed, we propose a list of 10 relevant overarching dimensions and attempt to define workers’ experience in the domain of WFH remotely. This definition can be used as a tool by researchers aiming to assess the experience of workers in order to inform the design or redesign of the sociotechnical systems that enable WFH.

**Supplementary Information:**

The online version contains supplementary material available at 10.1007/s41347-022-00264-4.

## Introduction

Advancements in technologies and connectivity since the late 1990s have enabled the diffusion of distributed modalities of working outside the office (Bloom et al., 2015; Tagliaro & Ciaramella, 2016). Researchers refer to the same distributed modalities of working with different names (Bolisani et al., 2020), e.g., teleworking, smart working, long-distance working, remote working at home, and working outside of the office. In this article, we focus only on working situations in which employees work outside of the office via a form of technology in their own adaptable space; therefore, we use the term “working from home” (WFH) to indicate a subpopulation of remote workers by aiming to focus on the perceived experience of those homeworkers remotely connected with their institutions.

WFH practices are regarded by companies as ways of reducing employees’ workload, through giving them the possibility to work (with formal and informal arrangements) a certain amount of time outside of their typical work facility (Davidescu et al., 2020). They are also seen as a strategic approach whereby companies can reduce the costs associated with their physical infrastructure (Angelici & Profeta, 2020; Mazzucchelli, 2017; Tagliaro & Ciaramella, 2016).

The practice of WFH was quite consolidated, but only moderately diffused, before the COVID-19 pandemic. For instance, data regarding job conditions in the 27 countries of the European Union in early 2020 suggested that on average, only 12% of employees were already used to this modality of working (EUROSTAT, 2021). Some researchers (Barrero et al., 2021; Bloom et al., 2015) have suggested that this low diffusion of WFH is mainly owed to a stigma of shirking associated with this modality of working, whereby remote workers were perceived as less controllable in terms of their performance compared to onsite employees. Empirical data reviews have also suggested that remote workers are less likely to be promoted compared to onsite workers, and that remote working positions are less desirable to productive workers (Bloom et al., 2015; Harrington & Emanuel, 2020). Researchers agree that WFH options should be better designed so that this work modality can become more desirable to highly productive workers, maximizing the benefit to companies and the workforce alike (Bloom et al., 2015; Harrington & Emanuel, 2020). In fact, some evidence suggests that when implemented, WFH not only reduces infrastructure costs but also increases the workforce’s overall productivity (Angelici & Profeta, 2020; Barrero et al., 2021; Bloom et al., 2015; Bolisani et al., 2020).

With the onset of the pandemic, remote working became a necessity for many (Shao et al., 2021; Zhang et al., 2021). Indeed, to ensure the continuity of services, governments, companies and institutions worldwide suddenly adopted approaches and systems to enable people to work from home (Bonacini et al., 2021). For instance, WFH reached peaks of 40% in eurozone countries (Fana et al., 2020).

Despite these adversities, with differing degrees of efficiency and effectiveness, citizens all over the world have been offered a certain amount of continuity regarding key services. For instance, with regard to education, the United Nations (2020) has reported that during the height of the pandemic, about 80–85% of students in high-income countries were involved in alternative e-learning activities, compared to only 50% in low-income countries.

Some researchers have suggested that COVID-19 has exposed many workers to the advantages and disadvantages of WFH, potentially increasing their awareness of possible (alternative) work modalities and opening the opportunity to reduce the stigma around WFH (Barrero et al., 2021; Harrington & Emanuel, 2020). For instance, according to a recent global Ipsos survey involving 12,500 employees across 29 countries, 30% of respondents who have experienced WFH would consider looking for another job if requested to return full-time to their office (Boyon, 2021).

Certainly, during COVID-19, companies and workers have invested in physical, digital and human capabilities (Barrero et al., 2021) to adapt to the necessity of WFH. Moreover, rapid technological advancements have supported the switch to WFH, with new and updated digital systems for online and remote services developed to sustain services and activities (Renu, 2021—e.g., conference, team management and exchanges, applied robotics solutions, e-health, online entertainment) including conferences, team management and exchange, applied robotics solutions, e-health, and online entertainment.

The technological, human and organizational investments made during COVID-19 may increase the impetus to rethink work practices and workplaces already initiated before the pandemic crisis (Lund et al., 2021). In this sense, WFH whether full- or part-time is likely to become a new job condition for a significant fraction of workers worldwide. The portion of the workforce that will aim to operate according to the WHF modality is hard to predict, and ultimately the decision to implement and support it in private and public services will be determined by cost–benefit considerations and by looking at the indexes of productivity and performance (European Commission, 2020). To inform decisions regarding the implementation of the WFH modality, it is however important to go further than economics and performance considerations and to additionally consider the impact of WFH on individuals, in order to avoid its well-known negative effects, such as increased workload and stress (Mallia & Ferris, 2000; Troup & Rose, 2012), isolation and decreased socialization (Dolan, 2011; Morgan & Symon, 2002; Raffaele & Connell, 2016), lack of placeness (Riva et al., 2021), and work-life balance issues (Aczel et al., 2021). Ultimately, deciding to implement or expand practices of WFH should also be driven by the possibility to monitor, evaluate and compare the overall experience of employees under such new working conditions in order to optimize and customize these emerging sociotechnical systems (i.e., systems to exchange information and perform actions among humans, supported by technology and regulated by spatial, internal and external normative constraints) to the needs of both workers and employers (Fox, 1995).

## Experience of Working from Home Remotely

WFH is more than simply performing at home the work conventionally done onsite. It requires a different individual and organizational structure, which should be designed to fit the needs and the goals of employers and employees alike (Baruch, 2000). To understand how different modalities under the umbrella of WFH can be designed and compared, it is necessary to identify ways for assessing workers’ subjective experience (Baruch, 2000; Wang et al., 2021), with the aim of achieving sustainable WFH modalities (Davidescu et al., 2020). Indexes of performance and productivity, as well as the amount of time spent working from home in line with contractual or informal agreements (e.g., flexibility), only provide a partial picture of the impact of WFH modalities. Indeed, monitoring in an efficient way workers’ perceived quality in performing their work and their overall experience when at home is considered a key aspect for improving the success of this work modality, as perceived by workers (Aczel et al., 2021; Ipsen et al., 2020, 2021; van der Lippe & Lippényi, 2020).

Workers’ experience of WFH has been widely and inconsistently characterized. For instance, some researchers agree that the subjective experience of workers is a multifaced concept that can be measured by collecting a wide range of variables regarding aspects such as satisfaction, flexibility, and work-life balance (Angelici & Profeta, 2020; Barrero et al., 2021; Bloom et al., 2015; Bolisani et al., 2020; Davidescu et al., 2020). Accordingly, Grant et al. (2013), focusing on the psychological impact of remote technology in the context of WFH, have suggested that workers’ experience is affected by their perceived effectiveness and performance, their well-being and work-life balance, plus overarching elements such as their use of technology and their relationship with management. By contrast, Aczel et al. (2021) have suggested that modeling the experience of working from home necessitates attention to the negative and positive aspects of WFH compared to working onsite, considering aspects such as control over tasks, freedom, motivation and individual context (e.g., number of children at home), on top of perceived performance, time spent at work, well-being, and work-life balance. Alternatively, Davidescu et al. (2020) have suggested that the measurement of subjective experience should be mainly focused on collecting aspects such as satisfaction regarding one’s position and work flexibility, that is, assessing the interplay between an employee’s subjective reaction (satisfaction) to objective conditions such as flexibility and their working conditions.

Certainly, a worker’s satisfaction, conceptualized either as the positive emotional state resulting from an individual’s appraisal of their job or job experience (Kröll & Nüesch, 2019), or as a set of an individual’s attitudes toward or about a certain job (Ahmad et al., 2003), is a key aspect of the workers’ overall experience. Nevertheless, due to this vague characterization of the construct of satisfaction, this concept is often used by researchers in a very opportunistic way. Based on the focus of the researchers, the assessment of workers’ satisfaction could be limited to modeling satisfaction toward the type of position and salary, or be expanded to include, for instance, satisfaction with work arrangements, the workload, the workers’ satisfaction regarding the technological and management enablers available at their institutions, etc. In this sense, satisfaction assessment is measured often with different goals and items by making it difficult to compare insights regarding this subjective component of the workers’ experience.

In terms of this opportunistic approach to satisfaction assessment, the concept of satisfaction and experience are not clearly connected in the context of WFH. In other domains, such as service and product interaction, experience is defined by an international standard (ISO 9241–210, 2019), which strongly connects experience and satisfaction with other potential aspects for a subjective assessment of a service’s quality. Such a standardized definition can enable goals and measurements to be unified in the domain of service experience but is missing in the context of WFH.

Some researchers (Bentley et al., 2016; Rajanen & Rajanen, 2020) have suggested that people’s experience of complex sociotechnical systems is usually determined by a subjective reaction to objective aspects and constraints. Therefore, it seems plausible that the interplay between a subjective reaction and objective aspects is a determinant in the quality experienced by people in WFH modalities. Nevertheless, given that the multifaceted concept of working experience is not well characterized when attempting to assess the experience of working from home, researchers tend to focus on different elements of a sociotechnical system (e.g., work modality and constraints) using a wide range of methods (which are more or less qualitative) and questions (which are more or less validated), often making it difficult to compare results (Duxbury et al., 1992; Grant et al., 2019; Maruyama et al., 2009). Indeed, some researchers are quite positive about the effect of WFH modalities on people (e.g., Angelici & Profeta, 2020; Bloom et al., 2015), whereas others are less positive or even negative, highlighting these modalities’ detrimental impacts (e.g., Bellmann & Hübler, 2020; de Vries et al., 2019).

Recognizing this issue, standardized scales are emerging to assess in a comparable way the elements that may be associated with the experience of WFH, for instance: (i) the e-work life scale (Grant et al., 2019), which aims to assess elements associated with effectiveness, work-life balance, and well-being; (ii) the advantage and disadvantage scale proposed by Ipsen et al. (2021), which seeks to model the strengths and weaknesses of WFH. Moreover, validated scales developed for other objectives are often used to assess the experience of remote workers, for instance, the technostress scale (Ragu-Nathan et al., 2008), which aims to assess the stress induced by one’s interaction with technology (Molino et al., 2020); and the Utrecht work engagement scale, intended as a measure of fulfillment at work (UWES, Schaufeli et al., 2006), which is often also used as an indirect measure of satisfaction (Molino et al., 2020; Moretti et al., 2020).

As recently suggested by Grant et al. (2019), researchers investigating WFH consider different variables to model workers’ experience. Nevertheless, despite variations in the objects measured, it is possible to aggregate different variables according to overarching dimensions. For instance, researchers can focus on assessing specific variables such as stress, workload or exhaustion, but despite the diversity of measures available, the overall objectives of such investigations are oriented toward assessing the same overarching concept, i.e., the effect of WFH on people’s well-being. Currently, a mapping of the aspects that researchers deem important for assessing the experience of remote home workers, as well as how these aspects are usually assessed, is missing. In response, the research reported here aimed—in line with the general objective of scoping reviews (Munn et al., 2018)—to systematically map the most common approaches for measuring home workers’ experience. This mapping was also intended to clarify the concept of experience in the domain of WFH by proposing a qualitative reorganization of the overarching dimensions that can be measured to model workers’ experience. Our scoping review was performed in accordance with the Preferred Reporting Items for Systematic Reviews and Meta-Analyses extension for Scoping Reviews (PRISMA-ScR) approach (Tricco et al., 2018).

## Methods

### Study Design

The scoping review was performed on articles examining or attempting to model the quality of experience of home workers from the past 10 years. The checklist of the PRISMA-ScR was used to ensure alignment of the review process with the framework (Supplementary material 1).

### Definition of Terms

WFH is conceptualized in this study as a modality of working from a remote location—specifically the home—via technology, enabling employees to perform their activities in a smart and connected way. The experience of the worker is understood here as workers’ perceived satisfaction and quality in performing their work from home.

### Research Questions

By aiming to map the current practice of assessing WFH in terms of the methods and types of tools used, and to identify the most investigated aspects as well as potential overarching dimensions used to evaluate the experience of workers, this review sought to answer the following research questions:

1. What methodological approaches (e.g., survey, observation, interview) are usually applied to investigate the subjective experience of WFH?
2. What variables are usually investigated using these approaches?
3. What overarching dimensions emerge through grouping variables oriented to assessing similar relationships between workers and their work modalities?

We intended the first two questions to help us map the most common methods applied and the variables investigated by researchers to assess workers’ experience. Furthermore and in line with previous research, we assumed that the experience of home workers is a multifaceted concept, and so we attempted to aggregate variables oriented to assessing similar relevant aspects of employees’ experience by proposing potential dimensions for evaluating this concept.

### Eligibility Criteria

In the review, we included records that:

(i) Focused on the evaluation of the quality perceived by remote home workers regarding their modality of work and its associated constraints, by highlighting empirically or qualitatively the main aspects that should be measured, irrespective of the COVID-19 pandemic.
(ii) Discussed and measured the advantages and disadvantages of WFH or proposed validation of tools for the assessment of the workers’ experience.
(iii) Attempted to assess or compare the perceived experience of WFH and office work.

We excluded records that investigated remote working outside of the home or working from home without the support of technology, as well as studies focusing on:

(i) Either the impact of the COVID-19 pandemic on workers’ quality of life or health, or disruption or changes brought about by the transition to WFH, without reflecting on key factors that may affect workers’ experience.
(ii) Either surveying workers, modeling by data set review aspects that affect workers’ productivity, or reviewing types of job arrangements or other organizational constraints on WFH, with no focus on aspects that affect people’s experience of working from home; or with researchers simply discussing theoretically the potential connection between job arrangements and the experience of workers while focusing mainly on modeling the workers’ performance..
(iii) The economic or environmental sustainability of working from home, the business advantages and general economic benefits of implementing this work modality, or the sustainability of WFH in the family context, without any or with minimal reference to effects on experience or satisfaction.
(iv) The social consequences of smart working enforced by COVID-19, e.g., the gender gap, and coping strategies.
(v) Behavioral changes resulting from legislation, rules, management/leadership and team interplay with regard to working from home owing to the pandemic crisis.

### Search Strategy

Records were retrieved from two of the largest literature databases—Scopus and Web of Science—using the Boolean operators (AND/OR) to combine the following keywords (see Supplementary Material 2 for the search criteria): “Working from home,” “Smart working,” “Remote working,” “Satisfaction,” “Perceived satisfaction,” “Experience,” and “Perceived quality.” We searched only for English language articles, conferences and book chapters. We focused on the past 10 years, to collect information on recent advancements in measuring employees’ experience with the WFH modality.

### Record Categorization Strategy

The records were first classified in terms of type of study and number of participants.

Subsequently, each record was analyzed to list down the variables declared by their authors to be the object of the investigation, and tested with qualitative or reliable scales (see Supplementary Material 3 for a qualitative synthesis). For each record, we reported the type of items used for the investigation as follows: (i) *qualitative scales*, whereby the record used its own items to investigate the dimensions at hand; (ii) *adapted/validated scales*, whereby the record used items adapted from previous studies or standardized reliability scales to investigate the dimensions at hand; or (iii) *mixture scales*, whereby the record used a mixture of qualitative and adapted/validated items to investigate the dimensions at hand.

The list of variables was used to group aspects by similarity, producing a set of clustered overarching dimensions. For instance, despite the fact that studies focusing on, for instance, how much people feel they matter at work (Prihadi et al., 2021), typically use different measures from those investigating, for example, how much a person is engaged personally or by others in their work activity (Moretti et al., 2020), both aim to attain workers’ insights regarding the same relationship, i.e., between individuals and their job function and activities. Two authors of the present study (MLDF, SB) independently categorized by a grounded approach (Stern, 1980) the records in potential overarching dimensions when, despite using different measures, researchers aimed to inform similar relationships between the workers and their modality of work. Agreement between the two authors was achieved via discussion, and the final set of dimensions was discussed and approved by all the authors.

Finally, we investigated how the overarching dimensions we identified have typically been investigated by researchers in terms of qualitative and quantitative scales.

## Results

**Fig. 1:**
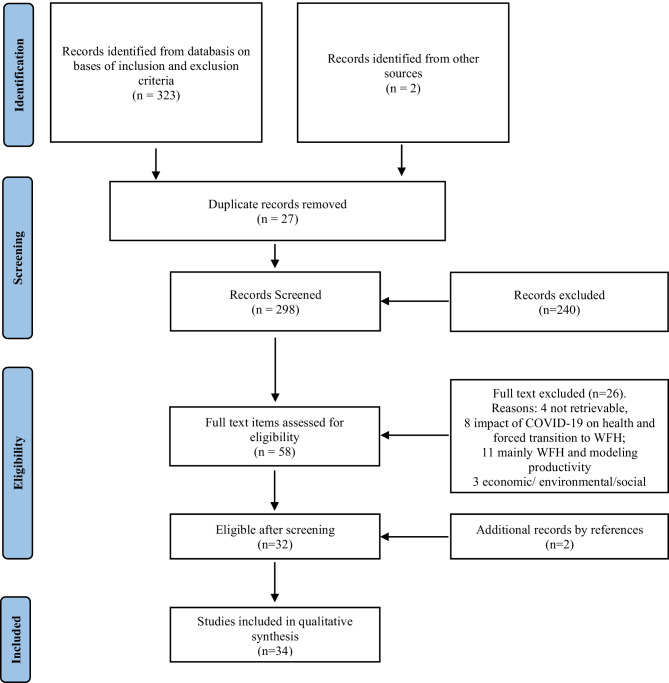
A pictorial view of the review process in accordance with the flowchart of the PRISMA guidelines (Liberati et al., 2009)

A total of 323 products were derived from Scopus and the Web of Science (Fig. 1). A further two records were added manually following a reading of online resources. After removing 27 duplicates, a scan of the 298 remaining records by title and abstract was performed by two of the authors (MLDF, SB). Articles that mentioned in their scope either a theoretical or empirical testing of aspects related to the quality experienced by workers in the context of remote WFH and that also suggested specific approaches or measures regarding this subject were retained.

Only about 19.5% (58) of the records were retained, as a large proportion of the studies were focused either on the economic and business advantages of implementing WFH, on the environmental sustainability of working from home, or on COVID-19-related aspects of WFH like the forced transition to this modality. The full texts of these 58 records were then scanned by three of the authors (MLDF, SB, GB) to identify articles proposing or discussing methods for assessing the subjective experience of WFH. After removing 26 records, a further two records were retrieved from the reference list. The final list comprised 34 records.

As shown in Table 1, 59% of the studies in our pool used surveys, whereas 15% used a theoretical analysis or a qualitative approach. In 29% of cases, a combination of a survey and either a qualitative approach or a validation of scales was used, while 18% of the studies involved longitudinal observations. Only one record (Bloom et al., 2015) proposed a randomized control experiment. The number of participants involved in the studies varied from 12 families to 11,011 individual participants.

**Table 1:** Type of research approach and number of participants in each study. Records are presented in ascending order by year

Study ID	Authors, year	Type of study	Number of participants
1	Eng et al. (2010)	Survey	1,103
2	Nansen et al. (2010)	Qualitative observation (longitudinal)	12 families (24 participants)
3	Wang and Ronen (2011)	Theoretical/review	N/A
4	Troup and Rose (2012)	Survey	856
5	Koopmans et al. (2013)*	Scale validation	1,181
6	Koopmans et al. (2014)*	Interview and survey	695 (expert interview and survey), 253 expert survey
7	Raguseo et al. (2014)	Interview and survey	100 (survey), 49 (interview)
8	Tustin (2014)	Focus group and survey	310
9	Bloom et al. (2015)	Survey and randomized experiment (longitudinal)	249
10	Malik et al. (2016)	Survey	117
11	Mazzucchelli (2017)	Interview and survey	1560 (workers), 160 (managers)
12	de Vries et al. (2019)	Diary study and survey (longitudinal)	61
13	Grant et al. (2019)	Scale validation	260
14	Nakrošienė et al. (2019)	Survey	128
15	Angelici and Profeta (2020)	Survey (longitudinal)	310
16	Bellmann and Hübler (2020)	Survey (longitudinal)	50
17	Bolisani et al. (2020)	Survey	931
18	Chong et al. (2020)	Survey	128
19	Davidescu et al. (2020)	Survey	220
20	Decastri et al. (2020)	Interview	57
21	Ipsen et al. (2020)	Scale validation	4643
22	Molino et al. (2020)	Survey and scale validation	1627
23	Moretti et al. (2020)	Survey	51
24	van der Lippe and Lippényi (2020)	Survey	11,011
25	Aczel et al. (2021)	Survey	704
26	Ali et al. (2021)	Survey	466
27	Craig et al. (2021)	Theoretical/review	N/A
28	Darouei and Pluut (2021)	Diary study and survey (longitudinal)	34
29	Di Tecco et al. (2021)	Survey	187
30	Ipsen et al. (2021)	Scale validation	5748
31	Langvik et al. (2021)	Survey	1,133
32	Negulescu and Doval (2021)	Theoretical/review	N/A
33	Prihadi et al. (2021)	Survey	400
34	Schade et al. (2021)	Survey	199

*Articles proposing relevant measures and aspects to assess workers’ experience not only of WFH modalities

Twenty-three percent of the studies only used a qualitative scale (e.g., individually defined items) for their analysis, 39% used scales with adapted items from previous literature or standardized scales, and 39% used a combination of qualitative and adapted questions.

As reported in Table 2, for each item we looked at which types of scales were used for the assessment, and we extrapolated 153 key variables reported by the researchers as part of their study as important for assessing the quality perceived by remote home workers (see Supplementary Material 3 for a qualitative synthesis of the measures used in the study). Moreover, 48% of the records reported the list of questions used to collect data, while only in 45% of the cases was the reliability of the items used for investigation discussed by the authors.

**Table 2:** Types of measurements (scales), items and reliability of items reported (yes/no) by the researchers, and key variables investigated in each study. Types of measurements are presented according to the following three categories: (i) qualitative scales—the record used its own items to investigate the dimensions at hand; (ii) adapted/validated scales—the record used items adapted from previous studies or standardized reliability scales to investigate the dimensions at hand; and (iii) mixture scales—the record used a mixture of qualitative and adapted/validated items to investigate the dimensions at hand. Review studies (Craig et al., 2021; Negulescu & Doval, 2021; Wang & Ronen, 2011) were excluded from the analysis of the types of items

Study ID	Authors, year	Type of scale	Items reported	Reliability reported	Key variables investigated
1	Eng et al. (2010)	Mixture	No	No	•Work and family conflict •Management support and influence
2	Nansen et al. (2010)	Qualitative	No	N/A	•Management of time and spatial constraints and conflicts
3	Wang and Ronen (2011)	N/A	N/A	N/A	•Loyalty toward company, peers and role •Job satisfaction
4	Troup and Rose (2012)	Mixture	Yes	No	•Living situation, including time spent on childcare (average hours per week) and distribution of work and home tasks •Performance •Job satisfaction
5	Koopmans et al. (2013)*	Adapted/validated	Yes	Yes	•Individual task and contextual performance •Counterproductive behavior
6	Koopmans et al. (2014)*	Adapted/validated	Yes	Yes	•Individual task and contextual performance •Counterproductive behavior
7	Raguseo et al. (2014)	Mixture	No	No	•Flexibility in the job •Management of work-life balance •Layout and technology elements •Innovativeness of management
8	Tustin (2014)	Mixture	Yes	No	•Advantages and disadvantages of WFH •Job satisfaction •Commuting duty and flexibility of the job •Work-life balance aspects, e.g., more time with family and better management of time •Well-being aspects, e.g., improved quality of life
9	Bloom et al. (2015)	Adapted/validated	Yes	Yes	•Performance •Commuting duty •Work-life situation •Satisfaction (life and work) •Exhaustion •Attitude toward work
10	Malik et al. (2016)	Mixture	Yes	Yes	•Perceived value of WFH •Family and work values and balance •Favorable attitude toward WFH •Motivational factors (intentions) •Organization of the work environment and job position
11	Mazzucchelli (2017)	Qualitative	No	No	•Family–work reconciliation •Flexibility •Lack of autonomy and support •Advantages and disadvantages
12	de Vries et al. (2019)	Mixture	Yes	No	•Engagement •Organizational commitment •Exchange with manager •Social isolation
13	Grant et al. (2019)	Adapted/validate	Yes	Yes	•Work-life interference •Flexibility •Well-being •Organizational aspects that affect WFH
14	Nakrošienė et al. (2019)	Qualitative	No	No	•Need to communicate with colleagues •Commuting and work-life balance e.g., taking care of family, WFH for sickness •Suitability of working space at home; •Supervisor’s trust and support •Access to organization’s documents •Time management and work home in productive hours •Satisfaction •Advantages of WFH •Self-reported productivity
15	Angelici and Profeta (2020)	Mixture	No	No	•Flexibility •Freedom of managing time and work activities •Subjective and objective productivity •Well-being •Work-life balance •Satisfaction
16	Bellmann and Hübler (2020)	Mixture	No	No	•Job satisfaction •Improved work-life balance •Workers personality •Job characteristics and organizational aspects •Commitment information •Collegiality of organization
17	Bolisani et al. (2020)	Adapted/validated	No	No	•Individual advantages and disadvantages of WHF
18	Chong et al. (2020)	Adapted/validated	No	No	•Stress •Exhaustion •Withdrawal behavior •Job satisfaction
19	Davidescu et al. (2020)	Qualitative	Yes	No	•Flexibility of job and time •Adaptability of working space organization and technology •Job satisfaction •Increased productivity and efficiency •Interpersonal relationships •Personal comfort and motivation •Management of working time
20	Decastri et al. (2020)	Qualitative	No	No	•Productivity •Management of work-life balance •Improved well-being of workers •Layout of the space and information and technology infrastructure •Quality of management and organization-related aspects
21	Ipsen et al. (2020)	Adapted/validated	Yes	Yes	•Advantages and disadvantages of WFH
22	Molino et al. (2020)	Adapted/validated	Yes	Yes	•Improved work-life balance •Stress in WFH •Stress induced by technology
23	Moretti et al. (2020)	Adapted/validated	No	Yes	•Engagement •Pain •Stress •Avoidance •Flexibility in tasks •Living situation •Perceived productivity •Advantages and disadvantages of WFH
24	van der Lippe and Lippényi (2020)	Mixture	No	Yes	•Work performance •Type of WFH oversight and collaboration •Perceived autonomy •Job satisfaction •Job demands •Job position •Situation at home, commuting and work-life balance
25	Aczel et al. (2021)	Qualitative	Yes	No	•Work efficiency •Well-being •Living situation and work-life balance •Advantages and disadvantages of WFH
26	Ali et al. (2021)	Qualitative	No	Yes	•Job satisfaction •Motivation •Organizational aspects •Personal fears and anxiety
27	Craig et al. (2021)	N/A	N/A	N/A	•Management of breaks and time •Management of elements in the work space/layout •Positive effect on well-being
28	Darouei and Pluut (2021)	Adapted/validated	No	Yes	•Engagement •Exhaustion •Attitude toward the organization •Work pressure/demands •Work-life conflicts
29	Di Tecco et al. (2021)	Mixture	Yes	Yes	•Engagement •Work-life balance •Job satisfaction •Well-being •Demands of and control over the work activity •Peer support •Management support •Rules and changes at the organizational level
30	Ipsen et al. (2021)	Adapted/validated	Yes	Yes	•Job satisfaction •Advantages and disadvantages of WFH •Perceived work-life balance •Perceived work efficiency •Perceived control overwork •Home office constraints •Work uncertainties •Inadequate tools
31	Langvik et al. (2021)	Mixture	No	No	•Personality •Job satisfaction •Stress •Socialization needs •Type of flexibility
32	Negulescu and Doval (2021)	N/A	N/A	N/A	•Time management •Space organization, setup and management
33	Prihadi et al. (2021)	Adapted/validated	Yes	Yes	•Mattering •Self-esteem •Extraversion •Work self-efficacy
34	Schade et al. (2021)	Mixture	Yes	Yes	•Work-related basic needs satisfaction •Job role •Autonomy and oversight •Support by colleagues •Well-being and exhaustion •Tendency to reappraise •Detachment from work •Flow of the work modality •Work engagement

Ten overarching dimensions were identified as follows:

1. Engagement with work (ENG): this dimension contains aspects investigated in 13 records (Bellmann & Hübler, 2020; Bloom et al., 2015; Chong et al., 2020; Darouei & Pluut, 2021; de Vries et al., 2019; Decastri et al., 2020; Di Tecco et al., 2021; Koopmans et al., 2013, 2014; Moretti et al., 2020; Prihadi et al., 2021; Schade et al., 2021; van der Lippe & Lippényi, 2020). These records assessed variables associated with workers’ individual relationships with their work in general, their work function and activities. This dimension also includes workers’ sense of fulfillment in doing their work, their sense of caring about their work and their perception that they matter, or their attitude and behavior toward their work that might affect (if negative) their willingness to work e.g., counterproductivity.
2. Flexibility (FLEX): This dimension contains aspects considered in 19 records (Angelici & Profeta, 2020; Bloom et al., 2015; Craig et al., 2021; Davidescu et al., 2020; de Vries et al., 2019; Decastri et al., 2020; Eng et al., 2010; Grant et al., 2019; Langvik et al., 2021; Mazzucchelli, 2017; Moretti et al., 2020; Nakrošienė et al., 2019; Nansen et al., 2010; Negulescu & Doval, 2021; Raguseo et al., 2014; Schade et al., 2021; Tustin, 2014; van der Lippe & Lippényi, 2020; Wang & Ronen, 2011). This dimension pertains to the assessment of variables such as the amount of flexibility in the job (e.g., working from home weekly) and perceived freedom to work at one’s own pace, i.e., time, space and resource management. This dimension also includes aspects related to the amount of oversight imposed on workers and perceived constraints on carrying out one’s work in a flexible way.
3. Health and well-being (HEAL): This dimension includes aspects presented in 16 records (Aczel et al., 2021; Angelici & Profeta, 2020; Bloom et al., 2015; Chong et al., 2020; Craig et al., 2021; Darouei & Pluut, 2021; Decastri et al., 2020; Di Tecco et al., 2021; Grant et al., 2019; Koopmans et al., 2013, 2014; Langvik et al., 2021; Molino et al., 2020; Moretti et al., 2020; Schade et al., 2021; Tustin, 2014). The variables investigated are related to the WFH modalities perceived effects on people’s health and well-being, for instance, fatigue, workload, stress (also induced by technology, Molino et al., 2020), pain, avoidance, and exhaustion induced by stress and withdrawal behavior.
4. Layout and technology (LAY). This dimension is apparent in six records (Craig et al., 2021; Davidescu et al., 2020; Decastri et al., 2020; Nakrošienė et al., 2019; Negulescu & Doval, 2021; Raguseo et al., 2014). The LAY dimension considers organizational and environmental elements that may affect workers’ experience, such as the physical adaptability of the workspace and furniture, and the adaptability and functionality of the technology, e.g., the quality of the technological setup, and issues in usage.
5. Organizational and job-related aspects (ORG): This dimension contains aspects from 11 records (Ali et al., 2021; Bellmann & Hübler, 2020; Darouei & Pluut, 2021; de Vries et al., 2019; Eng et al., 2010; Grant et al., 2019; Malik et al., 2016; Nakrošienė et al., 2019; Raguseo et al., 2014; Schade et al., 2021; Wang & Ronen, 2011). The ORG dimension is focused on the quality of the relationship between workers and their organization, including variables such as loyalty and trust toward the company e.g., uncertainty regarding the work, but also trust toward managers and colleagues, and support provided by management and colleagues that may compromise the aforementioned relationship. The ORG dimension also includes the assessment of job position, type of company, salary, and work demands and pressure, for instance, to model differences in workers’ experience.
6. Performance, productivity and efficiency (PERF): This dimension contains aspects investigated in 12 records (Aczel et al., 2021; Angelici & Profeta, 2020; Bloom et al., 2015; Chong et al., 2020; Koopmans et al., 2013, 2014; Moretti et al., 2020; Nakrošienė et al., 2019; Prihadi et al., 2021; Tustin, 2014; van der Lippe & Lippényi, 2020; Wang & Ronen, 2011). It contains quantitative performance measures including indexes, and reported data on increases in productivity, task complexity, time on task, and the number of goals achieved in a set period of time. Moreover, it includes subjective perceptions of performance, such as regarding the efficiency or productivity of workers.
7. Personal needs and style (PERS): This dimension appears in nine records (Ali et al., 2021; Bellmann & Hübler, 2020; Chong et al., 2020; de Vries et al., 2019; Langvik et al., 2021; Malik et al., 2016; Nakrošienė et al., 2019; Prihadi et al., 2021; Schade et al., 2021). It includes the assessment of individual needs and characteristics, personality traits and style, which may affect people’s activities at (and toward their) work, such as the need to interact/communicate with others, the need for socialization or comfort, security and support, and personal internal motivation to perform activities in a certain way and fear and anxiety to perform due to personal reasons and style.
8. Satisfaction (SAT): This dimension appears in 13 records (Ali et al., 2021; Angelici & Profeta, 2020; Bellmann & Hübler, 2020; Bloom et al., 2015; Davidescu et al., 2020; Decastri et al., 2020; Di Tecco et al., 2021; Langvik et al., 2021; Nakrošienė et al., 2019; Schade et al., 2021; Troup & Rose, 2012; Tustin, 2014; van der Lippe & Lippényi, 2020). It pertains to the explicit assessment of satisfaction as an individual’s overall sense of satisfaction with their work, or their satisfaction with its position and modality.
9. Subjective gain (SUBJ): This includes aspects measured in 9 records (Bolisani et al., 2020; Ipsen et al., 2020, 2021; Malik et al., 2016; Mazzucchelli, 2017; Moretti et al., 2020; Nakrošienė et al., 2019; Schade et al., 2021; Tustin, 2014). It pertains to the assessment of (economic) value, forms of personal gain (also caused by external factors) that may stimulate workers to carry out their work in a certain modality, including (perceived) improvements in the flow and quality of the work done, and the perceived advantages and disadvantages of the WFH modality.
10. Work-life balance (WLB): This dimension appears in 21 records (Angelici & Profeta, 2020; Bellmann & Hübler, 2020; Bloom et al., 2015; Decastri et al., 2020; Eng et al., 2010; Grant et al., 2019; Malik et al., 2016; Mazzucchelli, 2017; Nakrošienė et al., 2019; Nansen et al., 2010; Raguseo et al., 2014; Troup & Rose, 2012; Tustin, 2014). It contains aspects related to existing or perceived conflicts between work duties and duties associated with one’s living situation, e.g., home duty, childcare, distance from work, the necessity to commute, and the sustainability of working from home.

To summarize the relationship between the variables investigated in the records and the proposed overarching dimensions, we estimated the percentage of how many records focused on each dimension (see Supplementary Material 4). Specifically, the results suggest that aspects associated with WLB were the most investigated in our data set (62% of the records), followed by FLEX (56%) and HEAL (47%). Aspects concerning ENG and SAT were equally investigated (38%), followed by PERF (35%). Aspects associated with the other dimensions were investigated in fewer than one third of the records: ORG (32%), SUBJ and PERS (both 26%), and LAY (18%).

**Fig. 2:**
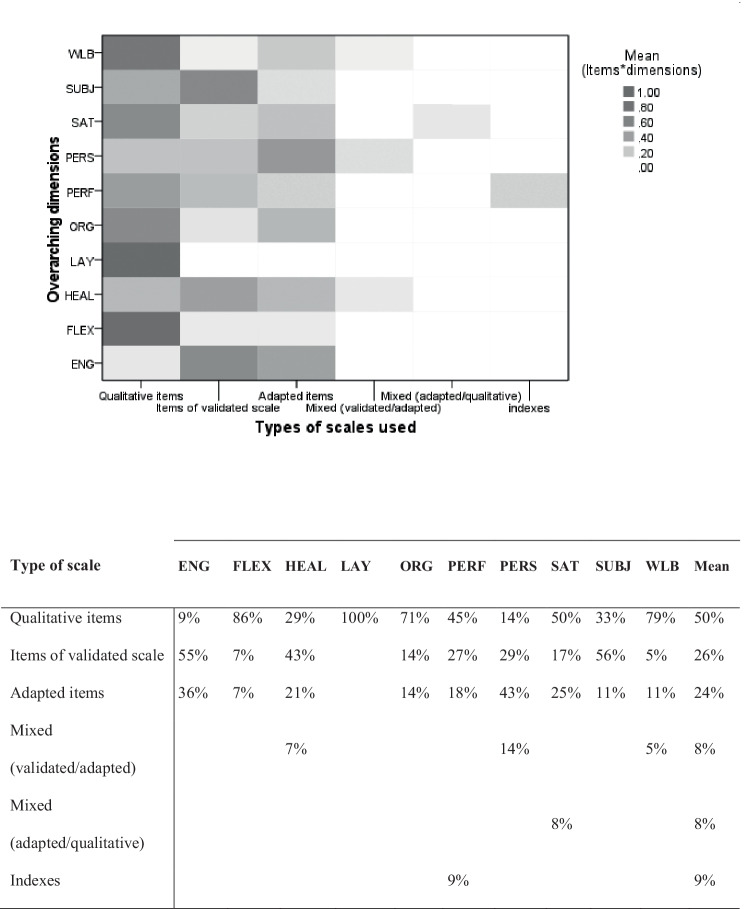
Heatmap of the types of scales (including indexes) reported in the records to assess workers’ experience by measuring aspects associated with the different overarching dimensions. Percentages are reported as proportions. Review studies (Craig et al., 2021; Negulescu & Doval, 2021; Wang & Ronen, 2011) were excluded from this analysis

To attain insights into how the overarching dimensions were investigated by the researchers, we distinguished for each dimension when data were collected using indexes, qualitative items, items of validated scales or adapted items, or a combination (mixture) of validated, adapted and qualitative items. We used this distinction to generate a heatmap in SPSS 25 (Fig. 2). As suggested in the figure, qualitative (own-made) items were widely used to measure all the clusters, with an overall mean of 50% of the dimensions measured by qualitative questions across the studies. These items were used in more than one third of the cases to assess aspects regarding LAY, FLEX, WLB, ORG, SAT, PERF, and SUBJ. Questions adapted from validated scales or previous studies were used to assess almost all the clusters (the exception being LAY) as on average between 24 and 26% of the dimensions in our records were measured with these types of questions. Items from validated scales were used to measure dimensions ENG, SUBJ, and HEAL in more than one third of the cases, while items adapted from previous studies (independently from the reliability of the questions) were often used to assess PERS and ENG. A combination of adapted and validated scale questions or indexes was rarely used to collect data associated with all the dimensions.

About 41% (14 out of 34) of the studies aimed at establishing the quality of the workers’ experience by comparing different modalities, e.g., WFH versus working at the office (Aczel et al., 2021; Ali et al., 2021; Angelici & Profeta, 2020; Bellmann & Hübler, 2020; Bloom et al., 2015; Davidescu et al., 2020; Ipsen et al., 2020, 2021; Mazzucchelli, 2017; Molino et al., 2020; Schade et al., 2021; Troup & Rose, 2012; Tustin, 2014; van der Lippe & Lippényi, 2020). While the remaining studies mainly focused on exploring specific aspects of WFH to model the experience of the workers without comparing it with office work. When researchers aim to compare working modalities by asking workers (from home and the office) to answer the same set of items, only a minimal focus is placed on the differences between the work modalities. For instance, when it comes to the dimension of SAT, researchers when comparing work modalities tend to assess workers’ general sense of satisfaction with their job. This valid measure of satisfaction is, however, different from assessing the workers’ satisfaction regarding specific elements of the work modality that is used by researchers who aim to assess the experience of WFH without comparative purposes, e.g., satisfaction with the quality of the setup and communication modalities, software used for WFH etc.

Due to the nature of their study (comparative or explorative of the work modalities), researchers may end up measuring different, albeit connected, aspects of workers’ experience. For instance, in 70% of the records in which the dimension of SAT was explored, researchers mainly refer to the workers’ general satisfaction with the job without focusing on the specificity of WFH settings (Ali et al., 2021; Angelici & Profeta, 2020; Bellmann & Hübler, 2020; Bloom et al., 2015; Davidescu et al., 2020; Schade et al., 2021; Troup & Rose, 2012; Tustin, 2014; van der Lippe & Lippényi, 2020). Conversely, in 30% of the cases, researchers did focus their analysis on satisfaction associated with the specific WFH settings to better understand how to improve the WFH modality and the effect of this modality on workers (Decastri et al., 2020; Di Tecco et al., 2021; Langvik et al., 2021; Nakrošienė et al., 2019), asking, for instance, about satisfaction regarding the working arrangement at home, the technical setup, or the satisfaction with the WFH experience.

## Discussion

The results of the qualitative synthesis reinforce the idea that workers’ subjective experience requires that multiple aspects be investigated. Below, we summarize the results in line with our research questions.

### Methodological Approaches That Researchers Commonly Apply to Investigate Workers’ Subjective Experience of WFH (R1)

Distributing questionnaires to workers represents the most commonly used approach for investigating workers’ subjective experience of WFH. Qualitative approaches such as interviews have also been used in combination with scales, either to focus on specific aspects or to model relationships among aspects that affect the experience. Purely qualitative studies and control experiments have less commonly been used to investigate the experience of WFH. Moreover, longitudinal observations are also performed, suggesting the idea that some researchers consider the experience of workers as an aspect that is changing and should be observed over time. This resonates with the definition of experience in other domains, such as the one of service and user experience (ISO 9241–210, 2019). Defining an international standard that could convey a cross-domain framework to guide researchers toward a unified evaluation practice and thus enabling comparability of the experience of remote workers, should be a long-term objective of the different communities that are interested in investigating WFH.

### Variables That Are Usually Investigated by Researchers (R2)

Despite the fact that the terminology used in different studies varies substantially, in many cases researchers have gathered—through diverse approaches, albeit mainly adopting validated scales—data to assess similar and connected key aspects. Out of the 153 variables declared by the researchers in our data set, in fewer than 50% of the cases were the items and the reliability reported. Our qualitative analysis of the records suggests that at least ten dimensions can affect the overall experience of working from home.

### Overarching Dimensions to Assess the Multiple Aspects Associated with Remote Workers’ Experience (R3)

The variables that we originally clustered as WLB and FLEX are the most commonly investigated, especially because these have also often been used to profile workers and their (contractual and living) conditions, as well as how these aspects affect their overall experience. From a subjective point of view, monitoring workers’ satisfaction regarding their work-life balance or the flexibility offered by their company may provide useful indicators for managers. For instance, sudden drops may imply issues with employees’ working modality and indicate that remedial actions are needed to maximize their experience. Other primary concerns for researchers are workers’ HEAL, ENG, and SAT, which could represent their subjective reactions to their working conditions, and declines in any of them may also affect their PERF. Performance can be observed using indexes (Bloom et al., 2015) or through measuring time spent on particular tasks. Furthermore, from a subjective point of view, focusing on workers’ perceptions of improved productivity (Nakrošienė et al., 2019) may provide information on the relationship between productivity and experience. Moreover, to monitor subjective perspectives regarding ORG and LAY—and thereby (changes in) the relationship between workers and their organization, or issues due to the technological or environmental setup—it may be useful to measure, for instance, loyalty toward the company or satisfaction with technology. Finally, it may be helpful to assess how the same sociotechnical system of working is perceived by people with different types of SUBJ and PERS, in order to provide insights into how best to design or alter a WFH modality that can suit different people. Combining all these dimensions may provide a full picture of workers’ experience and support decision making regarding the implementation or modification of WFH practices.

The goals of the researchers might affect how the dimensions are observed, e.g., comparing or exploring the work modalities. Researchers aiming to compare the experience of the workers from home and from the office could tend to assess general aspects associated with the ten dimensions; conversely, researchers who aim to explore the effect of WFH on workers’ experience tend to assess the dimensions focusing on the specific characteristics of WFH. These diverse goals may bring researchers to discuss the same dimensions collecting data that are inherently different and hard to compare.The dimensions we have identified have mainly been assessed using informal questions, supporting the idea that there is an increasing need for reliable and comparable ways of evaluating aspects connected to the experience of WFH (Duxbury et al., 1992). Looking at our data, this seems particularly relevant for aspects that belong to WLB and FLEX, as well as those related to SAT, PERF, ORG, and LAY. When it comes to the assessment of ENG, HEAL, and SUBJ, researchers can benefit from the use of validated scales or adapted questions from previous studies. Furthermore, aspects such as PERS and SAT may benefit from validated scales, although it seems that adapted items are generally preferred by researchers. The use of qualitative and informal questions to study workers’ experience is not a problem per se, but because the data collected and the aspects investigated in the domain of WFH are hard to compare, the risk is that decisions about implementing or changing WFH practices in companies and institutions will be made by overrating the importance or the risks of certain aspects based on partial insights regarding workers’ experience.

### A Tentative Definition of WFH Experience

The 10 dimensions that emerged from this analysis can be used to propose an original tentative unifying definition of workers’ experience as follows.

Home workers’ experience is a multifaceted concept, which may vary and should be monitored over time (Angelici & Profeta, 2020; Bellmann & Hübler, 2020; Bloom et al., 2015; Darouei & Pluut, 2021; de Vries et al., 2019; Nansen et al., 2010). It is affected by workers’ living situation (WLB, contractual (FLEX) constraints, well-being (HEAL), sense of engagement (ENG) and satisfaction (SAT) with their work, and perceived performance and productivity (PERF). Moreover, their relationship with their organization (ORG) and the physical organization of their work environment and technology (LAY) may negatively or positively affect their experience, together with personal differences in terms of subjective gain (SUBJ) or personal needs and style (PERS). All these aspects, when assessed and monitored over time, can provide a full picture of workers’ experience and support decision making regarding how best to implement or change WFH practices.

Such a definition of experience represents an original and inclusive perspective of the quality perceived by home workers to support decision making with regard to the design of the WFH modalities at a systemic level. Nakrošienė et al. (2019), focusing on WFH by taking a job demands-resources perspective, have recently proposed a list of 10 aspects that researchers should consider in their assessments of WFH: “time-planning skills, possibility to work during the most productive time, reduced time for communication with co-workers, possibility to work from home in case of sickness, supervisor’s trust; supervisor’s support, possibility to save on travel expenses, possibility to take care of family members, suitability of the working place at home and possibility to access the organization’s documents from home.”(Nakrošienė et al., 2019, p. 96). All these aspects are included in our dimensions (FLEX, WLB, PERS, SUBJ, ORG, SAT, LAY, WLB), although our analysis has also added aspects related to engagement (ENG) and health (HEAL), which have been widely investigated in the existing literature.

The main limitation of the present study is that we have proposed a set of overarching dimensions that emerged from our analysis of the literature. A future study should aim to extend or revise the dimensions we have proposed by involving experts and employee panels in interviews in order to find consensus. Nevertheless, despite this limitation, this scoping review has mapped approaches and measures of home workers’ experience, highlighted key aspects that are commonly investigated and suggested a potential unifying definition of remote workers’ experience.The topic of WFH is of interest to multiple domains, from industrial and business settings to healthcare and public administration. In the present review we did not consider the different and specific needs of each domain, and future studies should explore such contextual needs. Moreover, we did not focus on specific software architectures and management tools that could, for instance, impact the experience of the workers, nor did we compare different socio-technical systems developed or adapted to enable WFH.

Despite these limitations, the present work proposed a new integrated set of dimensions that could help researchers in different domains to look at WFH in terms of workers’ experience. This might, for instance, facilitate researchers usually focused on health aspects to also consider, and include in their assessment, dimensions that are normally considered more relevant by researchers in other domains (e.g., business-oriented investigation on WFH), and vice versa help researchers mainly oriented toward assessing the experience for improving the workflow and productivity to better consider health aspects. The current work proposed an initial framework that could enable cross-contamination between the needs and interests of different communities, paving the road for a unified assessment of WFH in different domains.

## Conclusion

An essential part of the “new normal” after the COVID-19 pandemic will be a revision of the modality of work (Bonacini et al., 2021). Certainly, we have learned that several tasks, if not certain jobs, can be done from home with a minimal amount of effort. Nevertheless, the shift toward this new normal needs to be monitored. Mistakes in the design of sociotechnical systems, such as working organizations, may go unseen until the consequences are perceivable e.g., the performance of some workers suddenly decreases, or many workers decide concurrently to leave their jobs. Continuous measurements and agreement on ways of assessing WFH are necessary to benchmark different modalities of remote working. This will facilitate the exchange and diffusion of best practices among companies and institutions, which will be especially important if working from home becomes a typical condition for a growing group of employees (Barrero et al., 2021). Therefore, it is necessary to unify the domain of WFH, which is currently characterized by differences in terminology, objectives and ways of assessing aspects associated with workers’ experience. Some standardized tools for measuring a set of aspects associated with workers’ experience are currently available (Grant et al., 2019; Ragu-Nathan et al., 2008; Schaufeli et al., 2006, 2017). However, the same cannot be said for either a battery of items with the potential to combine measurements of experience or a unified perspective regarding aspects that should be assessed.

This article has contributed to this effort to find a consolidated way of evaluating the experience of WFH by mapping the most commonly assessed aspects and how these are usually measured by researchers. Moreover, we have defined home workers’ experience based on insights from the literature. Although further validation is needed, such a definition and its elements should be intended as an initial driver to support researchers in different domains to fully account for the workers’ perspective when assessing WFH systems. Our list of dimensions can be used by experts as a checklist for establishing aspects to assess or monitor in order to improve workers’ experience of carrying out their profession from home. In this sense, the list of dimensions we proposed could be seen as a way to bridge the communities of researchers that are working in different domains, with the common goal of assessing the experience of home workers, pushing researchers to consider a more coherent and comparable set of dimensions for their investigations.

## Supplementary Information

Below is the link to the electronic supplementary material.

Supplementary file1 (DOCX 26 KB)

Supplementary file2 (DOCX 13 KB)

Supplementary file3 (DOCX 37 KB)

Supplementary file4 (DOCX 19 KB)
