# Supplementary material for: Approaches Adopted by Researchers to Measure the Quality of the Experience of People Working from Home: a Scoping Review
Source: J Technol Behav Sci. 2022 Jul 6;7(4):451–67. doi: 10.1007/s41347-022-00264-4 (PMC9261248; doi:10.1007/s41347-022-00264-4)
Supplement: Supplementary file 2 — Supplementary file2 (DOCX 13 KB) [file 41347_2022_264_MOESM2_ESM.docx]

# **Supplementary material 2**

Scopus Search 5 May 2021

( TITLE-ABS-KEY ( “Remote working” ) OR TITLE-ABS-KEY ( “Smart working” ) OR TITLE-ABS-KEY ( “working from home” ) AND TITLE-ABS-KEY ( “Experience” ) OR TITLE-ABS-KEY ( “Perceived quality” ) OR TITLE-ABS-KEY ( “Perceived satisfaction” ) OR TITLE-ABS-KEY ( “satisfaction” ) ) AND ( LIMIT-TO ( PUBSTAGE , “final” ) ) AND ( LIMIT-TO ( DOCTYPE , “ar” ) OR LIMIT-TO ( DOCTYPE , “cp” ) OR LIMIT-TO ( DOCTYPE , “ch” ) ) AND ( LIMIT-TO ( PUBYEAR , 2021 ) OR LIMIT-TO ( PUBYEAR , 2020 ) OR LIMIT-TO ( PUBYEAR , 2019 ) OR LIMIT-TO ( PUBYEAR , 2018 ) OR LIMIT-TO ( PUBYEAR , 2017 ) OR LIMIT-TO ( PUBYEAR , 2016 ) OR LIMIT-TO ( PUBYEAR , 2015 ) OR LIMIT-TO ( PUBYEAR , 2014 ) OR LIMIT-TO ( PUBYEAR , 2012 ) OR LIMIT-TO ( PUBYEAR , 2011 ) OR LIMIT-TO ( PUBYEAR , 2010 ) ) AND ( LIMIT-TO ( LANGUAGE , “English” ) )

Web of Science 5 May 2021

“Remote working” (Title) OR “smart working” (Title) OR “working from home”(Title) AND “experience”(Topic) AND “perceived quality”(Topic) AND “perceived satisfaction”(Topic) AND “satisfaction”

Document type: Articles or Proceedings papers or Book Review

Language: English
