# Supplementary material for: Approaches Adopted by Researchers to Measure the Quality of the Experience of People Working from Home: a Scoping Review
Source: J Technol Behav Sci. 2022 Jul 6;7(4):451–67. doi: 10.1007/s41347-022-00264-4 (PMC9261248; doi:10.1007/s41347-022-00264-4)
Supplement: Supplementary file 3 — Supplementary file3 (DOCX 37 KB) [file 41347_2022_264_MOESM3_ESM.docx]

# **Supplementary material 3**

Variables collected and the modalities of data collection for each record. The modalities are reported according to the following four categories: i) descriptive variables – e.g., data about participants’ individual characteristics used for descriptive or data analysis purposes; ii) qualitative items, iii) items from adapted scales, and iv) validated (standardized) scales and indexes used to measure the variables. Moreover, the following aspects are detailed if reported by the authors: number and description of the items, and reliability of the tools.

| **Study ID** | **Authors, year** | **Variables investigated** | **Measures/Scales for the investigation** |
| --- | --- | --- | --- |
|  |  |  |  |
| 1 | Eng et al. (2010) | - Work and family conflict - Management support and influence | DESCRIPTIVE: Sex, Age;  QUALITATIVE items: 1 item Extra home hours, 1 item to assess Days worked at home, 6 items on Work social support by family;  ADAPTED - reliability not reported for the items adapted: Organizational support 4 items from Eisenberger et al. (1986). Manager style using 9 items of the Multifactor Leadership Questionnaire (Muenjohn & Armstrong, 2008) adapted from (Frone et al., 1992) and (Carlson et al., 2000) |
| 2 | Nansen et al. (2010) | - Management of time and spatial constraints and conflicts | DESCRIPTIVE: Age, gender;  QUALITATIVE Items: not reported |
| 3 | Wang and Ronen (2011) | - Loyalty toward company, peers and role - Job satisfaction | - |
| 4 | Troup and Rose (2012) | - Living situation, including time spent on childcare (average hours per week) and distribution of work and home tasks - Performance - Job satisfaction | DESCRIPTIVE: Age, gender, type of work/role, full/part-time.  QUALITATIVE: work-life balance modeled by the living situation with items regarding the number of children or others to take care, and sharing duty. Own made additional items regarding satisfaction with taking care of children and shared duty at home. The reasoning behind items and reliability not reported.  STANDARDISED: a work attitude scale of 14 items (reliability: .87) was used to measure (Warr et al., 1979). |
| 5 | Koopmans et al. (2013)* | - Individual task and contextual performance - Counterproductive behavior | DESCRIPTIVE: Age, gender, education.  STANDARDISED: Proposed and tested a new version of a scale to assess Individual work performance (IWPS) composed of 14 items (reliability: between 0.7 and 0.8) including items regarding counterproductive behaviors |
| 6 | Koopmans et al. (2014)* | - Individual task and contextual performance - Counterproductive behavior | DESCRIPTIVE: Age, gender, education, type of work, and type of company. List of 317 indicators of work performances resulted from literature, survey, and interview. A second survey was used to define a final list of 23 indicators of work performance including items regarding counterproductive behaviors. |
| 7 | Raguseo et al. (2014) | - Flexibility in the job - Management of work-life balance - Layout and technology elements - Innovativeness of management | QUALITATIVE: The survey used 7 items (not reported) with multiple options were used to survey Information about Layout, ICT, management style, and flexibility. Interviews items investigating aspects regarding work-life balance and management of innovation were not reported. |
| 8 | Tustin (2014) | - Advantages and disadvantages of WFH - Job satisfaction - Commuting duty and flexibility of the job - Work-life balance aspects e.g., more time with family and better management of time - Well-being aspects e.g., improved quality of life | DESCRIPTIVE: Unclear collection of demographic data.  QUALITATIVE: Proposed an own made set of items (list of items is not provided) with reliability from 0.75 to 0.98. The reasoning behind the items is not provided or discussed. |
| 9 | Bloom et al. (2015) | - Performance - Commuting duty - Work-life situation - Satisfaction (life and work) - Exhaustion - Attitude toward work | DESCRIPTIVE: Age, gender, education income, monthly expenses with open questions were used for the profiling.  QUALITATIVE: Data about the daily commute in minutes and living situation are used to model work-life balance.  STANDARDISED: Indexes were used to assess performance. Positive and Negative Affect Schedule PANAS composed of 16 items (reliability: not reported) was used to assess attitudes (Clark & Tellegen, 1988).  ADAPTED: Adapted from the Burnout Inventory (Maslach & Jackson, 1981) 6 items were used to estimate work exhaustion intended here as an indirect measure of satisfaction (reliability unclear). A post-survey was also implemented, to further explore WFH, but items are not explained. |
| 10 | Malik et al. (2016) | - Perceived value of WFH - Family and work values and balance - Favorable attitude toward WFH - Motivational factors (intentions) - Organization of the work environment and job position | DESCRIPTIVE: Age, gender, gross annual income, weekly hours worked, education level completed, firm size (number of employees).  QUALITATIVE: Qualitative items were used to profile the type of organization and the job position about nature of the firm (e.g. private or state enterprise), who would be paying the user’s WFH cost, and if a formal or informal policy covering flexible work was in place at the respondent’s organization. Three items measured work commute – home-work distance (kilometers), time (minutes), and weekly cost (dollars) – and two items tapped SWH commute – home-WFH distance (kilometers) and time (minutes).  ADAPTED: 20 own made items based on previous literature reliability of from 0.82 to 0.94. The complete list of items is only partially provided. Items regarding the perceived value of WHF, family, and work value were adapted from multiple sources (Carlson et al., 2015; Sanchez et al., 2006; Sweeney & Soutar, 2001). Similarly, items regarding reasons and motivations for the WFH and items regarding the intention to use WFH came from multiple resources (Ajzen, 1991, 2001; Fishbein & Ajzen, 1972). |
| 11 | Mazzucchelli (2017) | - Family–work reconciliation - Flexibility - Lack of autonomy and support - Advantages and disadvantages | DESCRIPTIVE: Age, gender, education, Living situation, Size of the town of residence, Place of work, Travel time to work, Employment status, Problems making ends meet, Household, work and care load, Sharing tasks within the family, Further problems. Items are not reported.  QUALITATIVE: Proposed 26 own made items to measure subjective benefit from WFH, this includes work-life balance but the main focus was on subjective gain. Items were based on previous literature, items and reliability are not reported |
| 12 | de Vries et al. (2019) | - Engagement - Organizational commitment - Exchange with manager - Social isolation | DESCRIPTIVE: Sex, Age, Education, Supervisory position or not; Qualitative items: 1 item daily activity from home;  ADAPTED: 3 items about exchanges with management (Scandura & Graen, 1984), 4 items about Organizational commitment (Allen & Meyer, 1990), 9 items work engagement, adapted from Schaufeli et al. (2006). 4 items regarding professional isolation, adapted from(Golden et al., 2008). Items reported in supplementary material |
| 13 | Grant et al. (2019) | - Work-life interference - Flexibility - Well-being - Organizational aspects that affect WFH | DESCRIPTIVE: Age, gender, role, type of company. STANDARDISED: eWorklife Scale (EWL) validation composed of 17 items |
| 14 | Nakrošienė et al. (2019) | - Need to communicate with colleagues - Commuting and work-life balance e.g., taking care of family, WFH for sickness - Suitability of working space at home; - Supervisor’s trust and support - Access to organization’s documents - Time management and work home in productive hours - Satisfaction - Advantages of WFH - Self-reported productivity | DESCRIPTIVE: Age (in years) and marital status/ living situation.  QUALITATIVE: qualitative items were used to profile the organizational aspects such as Organizational tenure (in years) and type of work flexibility. Moreover, 16 own made items regarding ten key factors and carriers opportunities were asked but the reasoning behind the items and reliability are not reported. |
| 15 | Angelici and Profeta (2020) | - Flexibility - Freedom of managing time and work activities - Subjective and objective productivity - Well-being - Work-life balance - Satisfaction | DESCRIPTIVE: age, gender, education, disability, living situation, work activity per day in minutes.  STANDARDARDISED: Objective productivity reported by the workers' supervisor.  QUALITATIVE 69 items partially own made and for the well-being, some items were adapted from: British Household Panel Survey regarding well-being (Taylor et al., 1993). The reliability of the items was not reported. To assess subjective productivity, satisfaction, well-being perceived freedom, work-life balance |
| 16 | Bellmann and Hübler (2020) | - Job satisfaction - Improved work-life balance - Workers personality - Job characteristics and organizational aspects - Commitment information - Collegiality of organization | DESCRIPTIVE: Age, gender, education, nationality, tolerance to risk, type of job (permanent or not), and type of role, Working hours per week, Training in the last weeks, income.  ADAPTED: 23 own-made items to assess work-life balance, satisfaction job type and communication in the workplace, issues associated with the work and the company. Items are reported to be connected to previous literature (Dex & Bond, 2005; Song & Gao, 2020), but the connection is not clear, items are not reported and reliability is not presented.  STANDARDISED: The Big5 personality test used is not reported, nor referenced. |
| 17 | Bolisani et al. (2020) | - Individual advantages and disadvantages of WHF | DESCRIPTIVE: Age, gender, education, type of work, number of weekly days (Not all reported).  STANDARDISED: 23 items developed based on (Ipsen et al., 2020). Items and reliability are not discussed in the record. |
| 18 | Chong et al. (2020) | - Stress - Exhaustion - Withdrawal behavior - Job satisfaction | DESCRIPTIVE: Age, gender, nationality, sleep quality.  ADAPTED: 18 items based on previous literature. Items are partially provided but the reliability of the items was not discussed. Specifically, items were adapted to measure General task interdependence (Pearce & Gregersen, 1991) to model task complexity, from the supportive behavior scale (Trougakos et al., 2015) and from the disruptive work events scale (Zohar et al., 2003) to model need for support, and work withdrawal behavior scale (Spector et al., 2006) to ascertain aspects related to the relationship with work activity. Moreover, exhaustion items were adapted from the previous scale (Maslach & Jackson, 1981). |
| 19 | Davidescu et al. (2020) | - Flexibility of job and time - Adaptability of working space organization and technology - Job satisfaction - Increased productivity and efficiency - Interpersonal relationships - Personal comfort and motivation - Management of working time | DESCRIPTIVE: Age, gender, principal occupation, seniority within the company, Company size, Sector of activity, Legal status of the company.  QUALITATIVE: A set of 14 items with different types of options (dichotomous, multiple options, and five Likert-scale) about the type of contract flexibility, management of time, and layout. 3 own made items about job satisfaction, degree of salary satisfaction, and degree of satisfaction regarding working conditions. Items are reported but the reasoning behind and the validity of the item is not discussed. |
| 20 | Decastri et al. (2020) | - Productivity - Management of work-life balance - Improved well-being of workers - Layout of the space and information and technology infrastructure - Quality of management and organization-related aspects | DESCRIPTIVE: Type of company.  QUALITATIVE: items not reported |
| 21 | Ipsen et al. (2020) | - Advantages and disadvantages of WFH | DESCRIPTIVE: Age, gender, education, type of work;  STANDARDISED: 27 items are discussed and tested to test the advantages and disadvantages of WFH (reliability >0.8) |
| 22 | Molino et al. (2020) | - Improved work-life balance - Stress in WFH - Stress induced by technology | DESCRIPTIVE: Age, gender, education, type of work;  STANDARDISED: 11 Items of the Technostress Scale (Ragu-Nathan et al., 2008) were validated in the context of WFH (reliability between .81 and .91). The Copenhagen psychosocial scale (Kristensen et al., 2006) was used to assess stress (reliability: .86).  ADAPTED: 3 items (Reliability: 0.85 ) concerning workload were adapted from literature (Melin et al., 2014). 3 items were adapted from literature (De Simone et al., 2018; Kinnunen et al., 2006)to assess work-family (Reliability: .90). |
| 23 | Moretti et al. (2020) | - Engagement - Pain - Stress - Avoidance - Flexibility in tasks - Living situation - Perceived productivity - Advantages and disadvantages of WFH | DESCRIPTIVE: Age, gender, weight, height, education, job levels,  QUALITATIVE: living situation, remote working experience, kind of job and its differences from traditional work (12 items not reported). Moreover, additional questions about productivity were not reported.  ADAPTED: 4 items from the Brief Pain Inventory reliability: 0.75 (Caraceni et al., 1996). 16 items of the Fear Avoidance Beliefs Questionnaire, reliability:0.8 (Monticone et al., 2012).  STANDARDISED: 17 items of the Utrecht Work Engagement Scale, reliability: .80 -.90 (Schaufeli et al., 2006); 16 items of the Fear Avoidance Beliefs Questionnaire, reliability:0.8 (Monticone et al., 2012). |
| 24 | van der Lippe and Lippényi (2020) | - Work performance - Type of WFH oversight and collaboration - Perceived autonomy - Job satisfaction - Job demands - Job position - Situation at home, commuting and work-life balance | DESCRIPTIVE: Age, gender, years of education, type of work, living situation, commuting time in hours, amount of work from home per week, time for tenure.  QUALITATIVE: three items multiple choice regarding the working from home activity in teams or alone. Own made items to assess productivity and job satisfaction were are not reported or discussed in terms of reliability.  ADAPTED: Individual work performance scale (IWPS) but it is not clear how many items they used from this battery, the reliability was reported equal to .85 (Koopmans et al., 2013). 4 items adapted Job Control Inventory (Karasek Jr, 1979) to assess autonomy (reliability: .86). 4 items adapted value commitment battery (Angle & Perry, 1981) to assess commitment (reliability: .75). 4 items adapted from the Job Content Questionnaire (Karasek et al., 1998) to assess physical job demands (reliability: .75). |
| 25 | Aczel et al. (2021) | - Work efficiency - Well-being - Living situation and work-life balance - Advantages and disadvantages of WFH | DESCRIPTIVE: Position, type of work, gender, age group, living situation and the age and the number of children, need for more efficient work from home.  QUALITATIVE: 15 efficiency or well-being related aspects of work were used to compare advantages and disadvantages of working from the office and home based on a (reported) previous pilot study. Two own made items regarding the ideal time that respondents want to spend WFH ) and feasibility of working from home. Items are presented, but the reliability of the items is not discussed. |
| 26 | Ali et al. (2021) | - Job satisfaction - Motivation - Organizational aspects - Personal fears and anxiety | DESCRIPTIVE: Gender, role, years of service, type of institution.  QUALITATIVE: unclear number of items regarding job motivation, effects of COVID, job satisfaction, and anxiety organizational issues the role of reimbursement and training. Reliablity:76 |
| 27 | Craig et al. (2021) | - Management of breaks and time - Management of elements in the work space/layout - Positive effect on well-being | - |
| 28 | Darouei and Pluut (2021) | - Engagement - Exhaustion - Attitude toward the organization - Work pressure/demands - Work-life conflicts | QUALITATIVE: Workplace. As part of the morning survey, respondents were asked to indicate whether they would work from home or at the office on that particular day.  ADAPTED: 5 items adapted from the work‐family conflict scale developed by Netemeyer et al. (1996). Reliability 0.93 -0.94.; Emotional exhaustion. Adapted six items from the emotional exhaustion subscale of the Maslach Burnout Inventory (Maslach & Jackson, 1981). Reliability α was 0.91. Positive and negative affect on the organization. Adapted Positive and Negative Affect Schedule (PANAS, Watson et al., 1988) Reliability: .69- .94. Three items to assess work pressure. Adapted from the five‐item workload scale previously used by Pluut et al. (2018). Reliability was 0.82.  STANDARDISED: Work engagement. The nine items (UWES; Schaufeli et al., 2006) Reliability 0.91 across days. Time pressure. Items are partially reported |
| 29 | Di Tecco et al. (2021) | - Engagement - Work-life balance - Job satisfaction - Well-being - Demands of and control over the work activity - Peer support - Management support - Rules and changes at the organizational level | DESCRIPTIVE: Gender, age, education, marital status, number of children aged less than 12 years, job seniority, and commuting.  QUALITATIVE: 1 Own made item was used to assess overall satisfaction towards the smart working experience. 4 own made items were used to assess the work-life balance  ADAPTED: 1 item based on the World Health Organization index (Topp et al., 2015) was used to assess well-being (reliability .89 -.90). 1 item from literature (Cortese & Quaglino, 2006) was used to assess satisfaction.  STANDARDISED: 18 items from Management Standards Indicator Tool were proposed in its Italian version, the reliability was indicated between .79 and .9. However, the validation study of this version is not reported. 3 items, from Ultra-Short Measure for Work Engagement (Schaufeli et al., 2017) were used to assess engagement (reliability: .71 - .78). |
| 30 | Ipsen et al. (2021) | - Job satisfaction - Advantages and disadvantages of WFH - Perceived work-life balance - Perceived work efficiency - Perceived control overwork - Home office constraints - Work uncertainties - Inadequate tools | DESCRIPTIVE: Gender, age, education, nationality.  STANDARDISED: 27 items were tested and presented with reliability from 0.55 to 0.72, items exclusion is discussed composing a solution with 23 items to assess advantages and disadvantages in WFH. The reliability of the 23 items solution was not discussed. |
| 31 | Langvik et al. (2021) | - Personality - Job satisfaction - Stress - Socialization needs - Type of flexibility | DESCRIPTIVE: Age group, gender, work situation/flexibility, living situation.  QUALITATIVE: 2 items own made regarding socialization and needs. The validity of the items and their adaptation were not discussed  STANDARDISED: Big Five model of the personality of 20 items ((John & Srivastava, 1999). ADAPTED: 1 item associated with stress (Elo et al., 2003). |
| 32 | Negulescu and Doval (2021) | - Time management - Space organization, setup and management | - |
| 33 | Prihadi et al. (2021) | - Mattering - Self-esteem - Extraversion - Work self-efficacy | DESCRIPTIVE: Age, gender, nationality, occupation. The origin of the items is reported, but it is unclear how items were modified and if all the original items were used.  ADAPTED: Authors used: the State Self-Esteem Scale, reliability:.73-.81 (Heatherton & Polivy, 1991). General Mattering Scale, reliability:.82 - .92 (Marcus & Rosenberg, 1987). Work self-efficacy was measured using items from the Remote Work Self Efficacy measure, reliability:.84 (Staples et al., 1999).  STANDARDISED: Extraversion scale of Big Five Inventory (John & Srivastava, 1999). |
| 34 | Schade et al. (2021) | - Work-related basic needs satisfaction - Job role - Autonomy and oversight - Support by colleagues - Well-being and exhaustion - Tendency to reappraise - Detachment from work - Flow of the work modality - Work engagement | DESCRIPTIVE: Gender, age, Qualitative: items regarding the type of flexibility, household size, working average hours per week, years on the work, appropriateness of work at home, living situation, and home duties.  STANDARDISED: 23 items of the work-Related Basic Needs satisfaction scale, reliability: .81 -.85 (Van den Broeck et al., 2010). The 20 items PANAS (Watson et al., 1988) was used to assess attitude and detachment together with 4 items from the Recovery Experience Questionnaire (Sonnentag & Fritz, 2007). 4 items were used to capture the flow of working from the flow short scale (Rheinberg et al., 2003). Work Engagement was assessed by the 9 items version of the Utrecht Work Engagement questionnaire, with reliability: .80 -.90 (Schaufeli et al., 2006). 6 items own made items on perceived clarity of the work (reliability not reported) |

**References of supplementary material 2**

Ajzen, I. (1991). The theory of planned behavior. *Organizational behavior and human decision processes*, *50*(2), 179-211. https://doi.org/10.1016/0749-5978(91)90020-T

Ajzen, I. (2001). Nature and operation of attitudes. *Annual review of psychology*, *52*(1), 27-58. https://doi.org/10.1146/annurev.psych.52.1.27

Allen, N. J., & Meyer, J. P. (1990). The measurement and antecedents of affective, continuance and normative commitment to the organization. *Journal of occupational Psychology*, *63*(1), 1-18. https://doi.org/10.1111/j.2044-8325.1990.tb00506.x

Angle, H. L., & Perry, J. L. (1981). An empirical assessment of organizational commitment and organizational effectiveness. *Administrative science quarterly*, *26*(1), 1-14. https://doi.org/10.2307/2392596

Caraceni, A., Mendoza, T. R., Mencaglia, E., Baratella, C., Edwards, K., Forjaz, M. J., Martini, C., Serlin, R. C., De Conno, F., & Cleeland, C. S. (1996). A validation study of an Italian version of the Brief Pain Inventory (Breve Questionario per la Valutazione del Dolore). *Pain*, *65*(1), 87-92. https://doi.org/10.1016/0304-3959(95)00156-5

Carlson, D. S., Kacmar, K. M., & Williams, L. J. (2000). Construction and initial validation of a multidimensional measure of work–family conflict. *Journal of vocational behavior*, *56*(2), 249-276. https://doi.org/10.1006/jvbe.1999.1713

Carlson, J., Rosenberger III, P. J., & Rahman, M. M. (2015). Cultivating group-oriented travel behaviour to major events: assessing the importance of customer-perceived value, enduring event involvement and attitude towards the host destination. *Journal of marketing management*, *31*(9-10), 1065-1089. https://doi.org/10.1080/0267257X.2015.1035309

Clark, L. A., & Tellegen, A. (1988). Development and validation of brief measures of positive and negative affect: The PANAS scales. *Journal of personality and social psychology*, *54*(6), 1063-1070. https://doi.org/10.1037//0022-3514.54.6.1063

Cortese, C. G., & Quaglino, G. P. (2006). The measurement of job satisfaction in organizations: A comparison between a facet scale and a single-item measure. *TPM-Testing, Psychometrics, Methodology in Applied Psychology*.

De Simone, S., Agus, M., Lasio, D., & Serri, F. (2018). Development and validation of a measure of work-family interface. *Journal of Work and Organizational Psychology*, *34*(3), 169-179. https://doi.org/10.5093/jwop2018a19

Dex, S., & Bond, S. (2005). Measuring work-life balance and its covariates. *Work, employment and society*, *19*(3), 627-637. https://doi.org/10.1177/0950017005055676

Eisenberger, R., Huntington, R., Hutchison, S., & Sowa, D. (1986). Perceived organizational support. *Journal of Applied Psychology*, *71*(3), 500-507. https://doi.org/10.1037/0021-9010.71.3.500

Elo, A.-L., Leppänen, A., & Jahkola, A. (2003). Validity of a single-item measure of stress symptoms. *Scandinavian journal of work, environment & health*, *29*(6), 444-451. https://doi.org/10.5271/sjweh.752

Fishbein, M., & Ajzen, I. (1972). Attitudes and opinions. *Annual review of psychology*, *23*(1), 487-544. https://doi.org/10.1146/annurev.ps.23.020172.002415

Frone, M. R., Russell, M., & Cooper, M. L. (1992). Antecedents and outcomes of work-family conflict: testing a model of the work-family interface. *Journal of Applied Psychology*, *77*(1), 65. https://doi.org/10.1037/0021-9010.77.1.65

Golden, T. D., Veiga, J. F., & Dino, R. N. (2008). The impact of professional isolation on teleworker job performance and turnover intentions: does time spent teleworking, interacting face-to-face, or having access to communication-enhancing technology matter? *Journal of Applied Psychology*, *93*(6), 1412. https://doi.org/10.1037/a0012722

Heatherton, T. F., & Polivy, J. (1991). Development and validation of a scale for measuring state self-esteem. *Journal of personality and social psychology*, *60*(6), 895. https://doi.org/10.1037/0022-3514.60.6.895

John, O. P., & Srivastava, S. (1999). *The Big-Five trait taxonomy: History, measurement, and theoretical perspectives* (Vol. 2). University of California Berkeley.

Karasek Jr, R. A. (1979). Job demands, job decision latitude, and mental strain: Implications for job redesign. *Administrative science quarterly*, *24*(2), 285-308. https://doi.org/10.2307/2392498

Karasek, R., Brisson, C., Kawakami, N., Houtman, I., Bongers, P., & Amick, B. (1998). The Job Content Questionnaire (JCQ): an instrument for internationally comparative assessments of psychosocial job characteristics. *Journal of occupational health psychology*, *3*(4), 322. https://doi.org/10.1037//1076-8998.3.4.322

Kinnunen, U., Feldt, T., Geurts, S., & Pulkkinen, L. (2006). Types of work‐family interface: Well‐being correlates of negative and positive spillover between work and family. *Scandinavian journal of psychology*, *47*(2), 149-162. https://doi.org/10.1111/j.1467-9450.2006.00502.x

Koopmans, L., Bernaards, C., Hildebrandt, V., van Buuren, S., Van der Beek, A. J., & de Vet, H. C. (2013). Development of an individual work performance questionnaire. *International journal of productivity and performance management*, *62*(1), 6-28. https://doi.org/10.1108/17410401311285273

Kristensen, H. Hannerz, A. Hogh, & Borg, V. (2006). The Copenhagen psychosocial questionnaire (COPSOQ). A tool for the assessment and improvement of the psychosocial work environment. *Scand. J. Work Environ. Health*, *31*(6), 438-449. https://doi.org/10.5271/sjweh.948

Marcus, F., & Rosenberg, M. (1987). *Mattering: its measurement and significance in everyday life* [Paper presentation]. Eastern Sociological Society Meetings, Annual meeting of the Eastern Sociological Association, Cincinnati, OH.

Maslach, C., & Jackson, S. E. (1981). *Maslach burnout inventory: research edition; manual*. Consulting psychologists press.

Melin, M., Astvik, W., & Bernhard-Oettel, C. (2014). New work demands in higher education. A study of the relationship between excessive workload, coping strategies and subsequent health among academic staff. *Quality in Higher Education*, *20*(3), 290-308. https://doi.org/10.1080/13538322.2014.979547

Monticone, M., Baiardi, P., Bonetti, F., Ferrari, S., Foti, C., Pillastrini, P., Rocca, B., Vanti, C., & Zanoli, G. (2012). The Italian version of the Fear-Avoidance Beliefs Questionnaire (FABQ-I): cross-cultural adaptation, factor analysis, reliability, validity, and sensitivity to change. *Spine*, *37*(6), E374-E380. https://doi.org/10.1097/BRS.0b013e31822ff5a7

Muenjohn, N., & Armstrong, A. (2008). Evaluating the structural validity of the multifactor leadership questionnaire (MLQ), capturing the leadership factors of transformational-transactional leadership. *Contemporary management research*, *4*(1), 3-14. https://doi.org/10.7903/cmr.704

Netemeyer, R. G., Boles, J. S., & McMurrian, R. (1996). Development and validation of work–family conflict and family–work conflict scales. *Journal of Applied Psychology*, *81*(4), 400-410. https://doi.org/10.1037/0021-9010.81.4.400

Pearce, J. L., & Gregersen, H. B. (1991). Task interdependence and extrarole behavior: A test of the mediating effects of felt responsibility. *Journal of Applied Psychology*, *76*(6), 838–844. https://doi.org/10.1037/0021-9010.76.6.838

Pluut, H., Ilies, R., Curşeu, P. L., & Liu, Y. (2018). Social support at work and at home: Dual-buffering effects in the work-family conflict process. *Organizational behavior and human decision processes*, *146*(May), 1-13. https://doi.org/10.1016/j.obhdp.2018.02.001

Ragu-Nathan, T., Tarafdar, M., Ragu-Nathan, B. S., & Tu, Q. (2008). The consequences of technostress for end users in organizations: Conceptual development and empirical validation. *Information Systems Research*, *19*(4), 417-433. https://doi.org/10.1287/isre.1070.0165

Rheinberg, F., Vollmeyer, R., & Engeser, S. (2003). [The assessment of flow experience] Die erfassung des flow-erlebens. In J. Stiensmeier-Pelster & F. Rheinberg (Eds.), *[Diagnosis of Motivation and Self-Concept] In Diagnostik von SelbstkonzeptLernmotivation und Selbstregulation* (pp. 262–279). Hogrefe.

Sanchez, J., Callarisa, L., Rodriguez, R. M., & Moliner, M. A. (2006). Perceived value of the purchase of a tourism product. *Tourism management*, *27*(3), 394-409. https://doi.org/10.1016/j.tourman.2004.11.007

Scandura, T. A., & Graen, G. B. (1984). Moderating effects of initial leader–member exchange status on the effects of a leadership intervention. *Journal of Applied Psychology*, *69*(3), 428. https://doi.org/10.1037/0021-9010.69.3.428

Schaufeli, W. B., Bakker, A. B., & Salanova, M. (2006). The measurement of work engagement with a short questionnaire: A cross-national study. *Educational and psychological measurement*, *66*(4), 701-716. https://doi.org/10.1177/0013164405282471

Schaufeli, W. B., Shimazu, A., Hakanen, J., Salanova, M., & De Witte, H. (2017). An ultra-short measure for work engagement. *European Journal of Psychological Assessment*, *35*(4), 577-591. https://doi.org/10.1027/1015-5759/a000430

Song, Y., & Gao, J. (2020). Does telework stress employees out? A study on working at home and subjective well-being for wage/salary workers. *Journal of Happiness Studies*, *21*(7), 2649-2668. https://doi.org/10.1007/s10902-019-00196-6

Sonnentag, S., & Fritz, C. (2007). The Recovery Experience Questionnaire: development and validation of a measure for assessing recuperation and unwinding from work. *Journal of occupational health psychology*, *12*(3), 204-221. https://doi.org/10.1037/1076-8998.12.3.204

Spector, P. E., Fox, S., Penney, L. M., Bruursema, K., Goh, A., & Kessler, S. (2006). The dimensionality of counterproductivity: Are all counterproductive behaviors created equal? *Journal of vocational behavior*, *68*(3), 446-460. https://doi.org/10.1016/j.jvb.2005.10.005

Staples, D. S., Hulland, J. S., & Higgins, C. A. (1999). A self-efficacy theory explanation for the management of remote workers in virtual organizations. *Organization Science*, *10*(6), 758-776. https://doi.org/10.1287/orsc.10.6.758

Sweeney, J. C., & Soutar, G. N. (2001). Consumer perceived value: The development of a multiple item scale. *Journal of retailing*, *77*(2), 203-220. https://doi.org/10.1016/S0022-4359(01)00041-0

Taylor, M. F., Brice, J., Buck, N., & Prentice-Lane, E. (1993). *British Household Panel Survey user manual: Volume A: Introduction, technical report and appendices*. University of Essex Colchester.

Topp, C. W., Østergaard, S. D., Søndergaard, S., & Bech, P. (2015). The WHO-5 Well-Being Index: a systematic review of the literature. *Psychotherapy and psychosomatics*, *84*(3), 167-176. https://doi.org/10.1159/000376585

Trougakos, J. P., Beal, D. J., Cheng, B. H., Hideg, I., & Zweig, D. (2015). Too drained to help: A resource depletion perspective on daily interpersonal citizenship behaviors. *Journal of Applied Psychology*, *100*(1), 227-236. https://doi.org/10.1037/a0038082

Van den Broeck, A., Vansteenkiste, M., De Witte, H., Soenens, B., & Lens, W. (2010). Capturing autonomy, competence, and relatedness at work: Construction and initial validation of the Work‐related Basic Need Satisfaction scale. *Journal of occupational and organizational psychology*, *83*(4), 981-1002. https://doi.org/10.1348/096317909X481382

Warr, P., Cook, J., & Wall, T. (1979). Scales for the measurement of some work attitudes and aspects of psychological well‐being. *Journal of occupational Psychology*, *52*(2), 129-148. https://doi.org/10.1111/j.2044-8325.1979.tb00448.x

Watson, D., Clark, L. A., & Tellegen, A. (1988). Development and validation of brief measures of positive and negative affect: the PANAS scales. *Journal of personality and social psychology*, *54*(6), 1063. https://doi.org/10.1037//0022-3514.54.6.1063

Zohar, D., Tzischinski, O., & Epstein, R. (2003). Effects of energy availability on immediate and delayed emotional reactions to work events. *Journal of Applied Psychology*, *88*(6), 1082-1093. https://doi.org/10.1037/0021-9010.88.6.1082
