# Supplementary material for: Approaches Adopted by Researchers to Measure the Quality of the Experience of People Working from Home: a Scoping Review
Source: J Technol Behav Sci. 2022 Jul 6;7(4):451–67. doi: 10.1007/s41347-022-00264-4 (PMC9261248; doi:10.1007/s41347-022-00264-4)
Supplement: Supplementary file 4 — Supplementary file4 (DOCX 19 KB) [file 41347_2022_264_MOESM4_ESM.docx]

# **Supplementary material 4**

Records associated with each dimension

| **Study ID** | **ENG** | **FLEX** | **HEAL** | **LAY** | **ORG** | **PERF** | **PERS** | **SAT** | **SUBJ** | **WLB** |
| --- | --- | --- | --- | --- | --- | --- | --- | --- | --- | --- |
| 1 |  | • |  |  | • |  |  |  |  | • |
| 2 |  | • |  |  |  |  |  |  |  | • |
| 3 |  | • |  |  | • | • |  |  |  |  |
| 4 |  |  |  |  |  |  |  | • |  | • |
| 5 | • |  | • |  |  | • |  |  |  |  |
| 6 | • |  | • |  |  | • |  |  |  |  |
| 7 |  | • |  | • | • |  |  |  |  | • |
| 8 |  | • | • |  |  | • |  | • | • | • |
| 9 | • | • | • |  |  | • |  | • |  | • |
| 10 |  |  |  |  | • |  | • |  | • | • |
| 11 |  | • |  |  |  |  |  |  | • | • |
| 12 | • | • |  |  | • |  | • |  |  |  |
| 13 |  | • | • |  | • |  |  |  |  | • |
| 14 |  | • |  | • | • | • | • | • | • | • |
| 15 |  | • | • |  |  | • |  | • |  | • |
| 16 | • |  |  |  | • |  | • | • |  | • |
| 17 |  |  |  |  |  |  |  |  | • |  |
| 18 | • |  | • |  |  | • | • |  |  |  |
| 19 |  | • |  | • |  |  |  | • |  |  |
| 20 | • | • | • | • |  |  |  | • |  | • |
| 21 |  |  |  |  |  |  |  |  | • |  |
| 22 |  |  | • |  |  |  |  |  |  | • |
| 23 | • | • | • |  |  | • |  |  | • | • |
| 24 | • | • |  |  |  | • |  | • |  | • |
| 25 |  |  | • |  |  | • |  |  |  | • |
| 26 |  |  |  |  | • |  | • | • |  |  |
| 27 |  | • | • | • |  |  |  |  |  |  |
| 28 | • |  | • |  | • |  |  |  |  | • |
| 29 | • |  | • |  |  |  |  | • |  | • |
| 30 |  |  |  |  |  |  |  |  | • |  |
| 31 |  | • | • |  |  |  | • | • |  | • |
| 32 |  | • |  | • |  |  |  |  |  |  |
| 33 | • |  |  |  |  | • | • |  |  |  |
| 34 | • | • | • |  | • |  | • | • | • | • |
| Total (%) | 38% | 56% | 47% | 18% | 32% | 35% | 26% | 38% | 26% | 62% |
